# Frequency-tagged EEG reveals category-selective responses to face race

**DOI:** 10.1093/scan/nsag059

**Published:** 2026-07-20

**Authors:** Pauline Schaller, Lisa Stacchi, Peter de Lissa, Roberto Caldara, Anne-Raphaëlle Richoz

**Affiliations:** Eye and Brain Mapping Laboratory (iBMLab), Department of Psychology, University of Fribourg, Fribourg, Switzerland; Eye and Brain Mapping Laboratory (iBMLab), Department of Psychology, University of Fribourg, Fribourg, Switzerland; Eye and Brain Mapping Laboratory (iBMLab), Department of Psychology, University of Fribourg, Fribourg, Switzerland; Eye and Brain Mapping Laboratory (iBMLab), Department of Psychology, University of Fribourg, Fribourg, Switzerland; Eye and Brain Mapping Laboratory (iBMLab), Department of Psychology, University of Fribourg, Fribourg, Switzerland

**Keywords:** race, face processing, other-race effect, face inversion effect, fast periodic visual stimulation

## Abstract

Racial-category information in faces is visually and socially salient, and is rapidly extracted by the human brain. Yet, its electrophysiological encoding remains unclear. Event-related potentials findings are mixed, reporting contradictory effects of race on neural responses. To inform this debate, we used fast periodic visual stimulation (FPVS), an alternative objective and implicit method to measure neural face categorization. In Experiment 1, Western European participants viewed a 6 Hz stream of same- or other-race faces with periodic oddball faces from the alternate race. Results showed no above-noise-level race discrimination responses. In Experiment 2, oddball faces were embedded in object streams, presented upright or inverted, to test whether race modulates face categorization. Neural face categorization responses were above noise level for both same- and other-race faces. Importantly, while upright stimuli showed similar responses to both races, inversion selectively reduced responses to same-race faces, resulting in stronger neural responses to other-race faces. These findings suggest that face inversion affects neural responses based on visual expertise with face categories. Overall, our results demonstrate that racial-category information is robustly indexed by FPVS, highlighting this method as a powerful neurofunctional tool to study social vision.

## Introduction

Human faces carry crucial social signals that play a central role in interpersonal communication and observers exhibit remarkable face processing proficiency in rapidly extracting a wide range of facial features to support various cognitive and social functions (Karnadewi and Lipp 2011, Rossion 2018, Young and Burton 2018, Blauch *et al*. 2021, Rodger *et al*. 2021, Richoz *et al*. 2024, Rodger and Caldara 2025). This expertise in face processing is, however, strongly influenced by an observer’s experience with specific categories of faces. Behaviorally, this is reflected in the differential processing of faces from the observer’s own race (i.e. same-race; SR) compared to those of other-races (OR). Two well-documented phenomena illustrate this effect: the Same-Race Recognition Advantage (SRRA), characterized by higher recognition accuracy for SR faces, and the Other-Race Categorization Advantage (ORCA), where race categorization is faster for OR faces. One of the main explanations for these phenomena is that perceptual expertise tunes the face processing system towards the most frequently encountered and socially relevant category (Meissner and Brigham 2001, Singh *et al*. 2022). While this enhanced specialized neural and cognitive processing increases SR face recognition, it has been hypothesized that it also slows down their categorization by race (Valentine 1991, Valentine and Endo 1992, Caldara and Abdi 2006, Ge *et al*. 2009). In contrast, OR faces, which are processed more coarsely, might allow faster access to racial information, thereby allowing for a faster categorization.

Race does not only influence face processing under typical viewing conditions, but it also modulates the occurrence of other well-established face-specific effects. One such phenomenon is the face inversion effect (FIE). The FIE refers to the well-documented decline in performance when faces are presented inverted rather than upright, an effect that is significantly stronger for faces compared to any other non-face object (Yin 1969, Albonico *et al*. 2018). While the majority of behavioral studies using face categorization by race tasks reported no race-related differences in the FIE (Caharel *et al*. 2011, Montalan *et al*. 2013, Wiese 2013, Colombatto and McCarthy 2017), those using face recognition or discrimination paradigms reported larger FIE for SR than OR faces, with the decline in face recognition accuracy being more pronounced for SR faces (Rhodes *et al*. 1989; Nachson and Catz 2003, Sangrigoli and De Schonen 2004, Hancock and Rhodes 2008, Megreya *et al*. 2011; but see Valentine and Bruce 1986). A prevailing explanation for this difference is rooted in how visual expertise shapes the face processing mechanisms. Holistic processing, which refers to the perception of a stimulus as a unified whole by relying on the configural integration of its features (i.e. the integration of spatial relationships between facial features), is more prominent for faces with which we possess extensive experience, such as SR faces (Tanaka et al., 2004, Michel *et al*., 2006a, Michel *et al*., 2006b, Hancock and Rhodes 2008). In contrast, the limited familiarity with OR faces leads to a greater reliance on piecemeal processing, wherein individual facial features are analyzed independently. As inversion disrupts the holistic perception of a stimulus (Farah *et al*. 1995, Rossion 2009), or more broadly impairs perceptual mechanisms optimized for frequently encountered stimuli (Murphy and Cook 2017, Gerlach and Mogensen 2024), it is more detrimental for SR faces (e.g. Caharel *et al*. 2011).

Although behavioral differences in the recognition and categorization by race of SR and OR faces are well established, there is limited consensus in the literature on whether, and how, these effects occur at the neural level. In the electrophysiological domain, several studies using event-related potentials (ERPs) have investigated the impact of face race on the N170 component–a face-sensitive negative deflection, peaking around 170 ms after stimulus onset and typically observed over posterior temporal sites (Bentin *et al*. 1996). Despite extensive investigation, findings remain inconclusive: while some studies have reported larger N170 amplitudes for upright SR than OR faces (e.g. Ito and Urland 2005, Senholzi and Ito 2013, de Lissa *et al*. 2024), others have found no race-related modulation of the N170 (e.g. Caldara *et al*. 2003, 2004, Stahl *et al*. 2010, Vizioli *et al*. 2010, Sun *et al*. 2013, Wiese 2013, Lv *et al*. 2015), or even a reversed pattern, with larger amplitudes for OR faces (e.g. Caharel *et al*. 2011, Montalan *et al*. 2013, Herzmann 2016, Spencer *et al*. 2018, Yao and Zhao 2019). Similar inconsistencies have been observed in how the neural FIE manifests across face races. For same-race faces, the neural FIE is typically characterized by increased amplitude and delayed latency of the N170 component for inverted faces (e.g. Wiese *et al*. 2009, Vizioli *et al*. 2010, Caharel *et al*. 2011, Hahn *et al*. 2012, Chen *et al*. 2013, Montalan *et al*. 2013, Wiese 2013, Tong *et al*. 2014, Allen-Davidian *et al*. 2021). As for SR vs. OR FIE differences, some studies revealed greater FIE for SR than OR faces (Vizioli *et al*. 2010, Wiese 2013), whereas others reported similar FIEs on the N170 component for both races (Wiese *et al*. 2009, Hahn *et al*. 2012, Chen *et al*. 2013, Tong *et al*. 2014, Zhou *et al*. 2015). Finally, one study reported the reverse pattern, with a larger FIE for OR than SR faces (Montalan *et al*. 2013). Such inconsistency in the literature may be attributed to a lack of precision, sensitivity or objectivity in the ERP measure. Specifically, different levels of attention or memory load may modulate the N170 (Morgan *et al*. 2008, Mohamed 2017). This component is also susceptible to noise, including artifacts from muscle activity (Achaibou *et al*. 2008, Sel *et al*. 2015). In addition, variability in latency across trials can reduce the precision of ERP measures (Guy *et al*. 2021, Yildirim-Keles *et al*. 2025). When responses are averaged, such variability may blur the neural signal, leading to less well-defined peaks. The low-level properties of the stimuli when not controlled could also modulate the ERPs responses. Furthermore, the objectivity of ERP analysis is often compromised by the subjective determination of filters (Zhang *et al*. 2024) or the timing, amplitude, and location of ERP components (Mahini *et al*. 2020), leading to variability in interpretation. Finally, the presentation of stimuli in isolation, as is common in ERP paradigms, raises questions about whether the recorded neural activity specifically reflects face processing or if it also encompasses more general visual processing responses. This highlights the need for an alternative methodology that can effectively address these limitations, or at least provide more insights to feed this debate.

Fast Periodic Visual Stimulation (FPVS) overcomes these constraints (Rossion 2014, Norcia *et al*. 2015). FPVS presents base stimuli interspersed with oddball stimuli at predefined frequencies, eliciting frequency-tagged neural responses. This paradigm relies on the assumption that if the brain can distinguish between base and oddball stimuli, it exhibits differential neural response synchronized to the two distinct frequency rates. This oddball-FPVS paradigm has extensively addressed face identity discrimination (Liu-Shuang *et al*. 2014, Stacchi *et al*. 2019, Rossion *et al*. 2020; Stacchi and Caldara 2022), recognition (Zimmermann *et al*. 2019), face-object categorization (de Heering and Rossion 2015, Rossion *et al*. 2015), and emotional expression discrimination (Dzhelyova *et al*. 2017, Coll *et al*. 2019, Van der Donck *et al*. 2020). FPVS enables objective frequency-domain quantification with high signal-to-noise ratio by isolating relevant processes to narrow frequency bands while noise spans a broader range (Liu-Shuang *et al*. 2014). Additionally, the rapid presentation prevents feature-by-feature processing and limits compensatory strategies (Dzhelyova *et al*. 2020), which might be otherwise used during inversion. Using varied natural stimuli within single sequences ensures responses are image-independent and mirrors real-life perceptual complexity (Zimmermann *et al*. 2019). Challenging conditions have been shown to reveal subtle differences (Ambadar *et al*. 2005, Bould and Morris 2008, Richoz *et al*. 2024), and might therefore also enhance SR versus OR processing differences. Altogether, the precision, objectivity, ecological validity, and complexity offered by the FPVS paradigm makes it well-suited to investigate the neural mechanisms underlying SR and OR face processing, as well as its interaction with well-known face-specific effects, such as the neural face inversion effect. In the face individuation domain, a recent FPVS study in 6- and 9-month-old infants found larger face-sensitive responses to SR than OR faces, along with significant face individuation responses for both races (Wallsinger *et al*. 2025). However, face race did not impact identity discrimination. The authors suggested that, at 6 months old, greater visuocortical resources are allocated to SR than OR faces, but race does not appear to modulate face individuation mechanisms per se. In adults, where the distinction between familiar SR and less familiar OR faces is typically more robust, race-related effects might be expected to emerge more strongly. However, to the best of our knowledge, FPVS has not yet been applied to investigate race processing in adults, nor has it been used to examine neural race discrimination or race-related modulation of face categorization.

To address this gap and further inform the debate on the neural sensitivity to face race, we recorded EEG from Western European participants across two oddball-FPVS experiments. Experiment 1 assessed upright race discrimination with faces of one race (Eastern Asian or Western European) as base stimuli and alternate-race faces as oddball stimuli. Experiment 2 assessed face-object categorization with oddball faces inserted into object streams, presented both upright and inverted. Participants performed an orthogonal fixation-monitoring task.

## Experiment 1—race discrimination

### Methods

#### Participants

Forty-four participants of Western European descent (five men) aged between 18 to 33 years old (*M *= 22.7, *SD *= 3.2) took part in both experiments. Participants reported having no close relatives or close friends of non-Western European origin and no more than six months of residence in countries where Western Europeans are not the majority. By restricting the sample to Western European observers, we avoided variability in cross-cultural contact and perceptual expertise. This homogeneity allowed us to maintain adequate statistical power within our sample-size constraints. Accordingly, the SR and OR labels are defined relative to this specific observer group (SR for Western European faces; OR for East Asian faces). All participants had normal or corrected-to-normal vision. The majority were undergraduate students at the University of Fribourg, Switzerland, who voluntarily participated in exchange for experimental credits. The remaining participants were relatives of the experimenters. None of the participants had any history of neurological or psychiatric disorders. Their informed consent was ensured prior to the experiments by the signature of a consent form explaining the primary objectives of the study. The local ethics committee of the Department of Psychology approved the study.

#### Materials

We used the same black and white Western European (WE, Same-Race) real human face images as employed in a previous oddball-FPVS paradigm (Yildirim-Keles *et al*. 2025). All images were 320 × 320 pixels and varied in rotation, background, age, gaze direction, and the presence of accessories (e.g., jewelry, scarves, beards, glasses). We complemented this dataset with an equivalent set of East Asian (EA, Other-Race) faces, matched to the WE faces in terms of visual and demographic characteristics. The SR and OR stimulus sets were identical to those described in Schaller *et al*. (2023), each comprising 36 faces per race group, with equal numbers of men and women (*N *= 18), a comparable number of frontal and profile faces (*N *= 19), and a similar distribution of gaze direction (17 towards, 19 away). Backgrounds were also matched (12 plain, 8 clear details, and 16 blurred details), as were age categories (1 child, 24 young adults, 6 middle-aged adults, and 5 older adults), attractiveness (9 highly attractive faces, 4 women; 27 faces of standard attractiveness, 14 women), and facial expressions (13 slightly smiling, 23 neutral). To quantify within-set physical homogeneity, we computed the mean pixel-wise Pearson correlation across all possible image pairs within each category (SR and OR). While the mean correlation differed statistically between SR (*M *= 0.066; *SD *= 0.19) and OR (*M *= 0.044; *SD *= 0.21) sets, *t*(1737.9) = 2.31, *P* = .021, this difference was negligible in magnitude (Cohen’s *d *= 0.11). As the absolute difference in mean correlation was minimal (0.02), the statistical difference is likely driven by the large number of pairwise comparisons (*N *= 1738 pairs), which provides extremely high statistical power to detect even minimal effects. All stimuli were equalized in terms of luminance and contrast with the Matlab SHINE toolbox with the default option (Willenbockel et al. 2010 ). They were all displayed with a scaling factor of 1.4 at the center of a gray screen and subtended a visual angle of approximately 6.6° horizontally and vertically from a 75 cm-screen distance.

The experiment was launched via Matlab (The MathWorks, Inc., 2017) with the use of the Psychophysics Toolbox (PTB-3; Brainard 1997, Kleiner *et al*. 2007) on a VIEWPixx/3D monitor (VPixx Technologies, Saint-Bruno, Canada), with a refresh rate of 120 Hz and a resolution of 1920 × 1080 pixels.

#### Procedure

All participants took part in the two experiments, beginning with the face race discrimination task, followed by the upright face categorization task, and finishing with the inverted face categorization task. This specific order was maintained across all participants and was chosen based on the very nature of the tasks. The race discrimination task was conducted first to ensure optimal alertness of the participants. Conversely, the inverted face categorization task, which involved different face perception from the typical one, was scheduled last.

**Figure 1:**
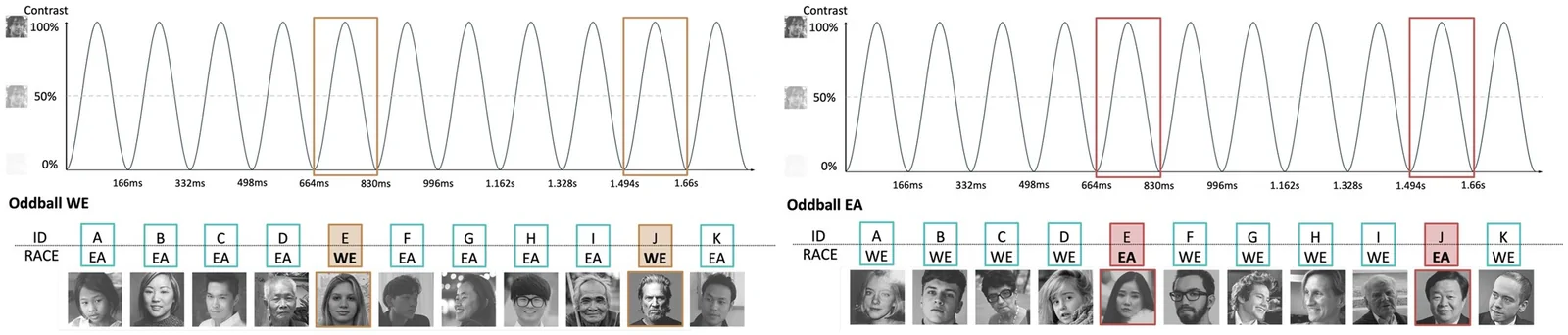
The paradigm of the oddball-FPVS race discrimination task. Note that the pictures provided are merely examples and do not represent an actual sequence of images.

More specifically for Experiment 1, each participant attended to four stimulation sequences with OR faces as oddball and four with SR faces as oddball, presented in a counterbalanced order (Fig. 1). The base stimuli were faces from the opposite race. Each stimulation sequence included 72 cycles consisting of four base stimuli followed by one oddball stimulus. Within each trial, stimuli presentation was semi-randomized; stimuli within a category (i.e. SR or OR faces) were presented in random order, with the constraint that no stimulus was repeated consecutively. The base and oddball frequencies were set at 6 Hz and 1.2 Hz, respectively, as these parameters have been shown to elicit significant face identity discrimination and categorization (Vettori *et al*. 2019, Zimmermann *et al*. 2019, Rossion *et al*. 2020, Hauk *et al*. 2021, Retter *et al*. 2021, Yildirim-Keles *et al*. 2025).

To avoid sudden stimulation onset and offset, 2 seconds of gradual fade-in and fade-out were added at the beginning and end of each trial. Stimuli were presented through sinusoidal contrast modulation. To maintain a constant level of attention during stimulation, participants were instructed to fixate on a central cross superimposed on the image and to press a button whenever the cross briefly (200 ms) changed color. This change occurred randomly 12 times per stimulation sequence.

During the experiment, participants were seated 75 cm away from a computer screen alone in an electrically shielded and soundproof room. Their head position was stabilized using a chin rest. A short pause was enforced every 4 trials. Experiment 1 lasted on average approximately 15 minutes.

#### EEG acquisition and preprocessing

Electrophysiological signals were recorded using a 128-channel Biosemi Active-Two (Amsterdam, Netherlands) with Ag/AgCl active electrodes and digitized with a sampling rate of 1024 Hz. Offset was measured in reference to the CMS and DRL electrodes (Driven Right Leg and Common Mode Sense, respectively) and it was reduced and maintained between +/- 25 μV throughout the whole testing session. Triggers were sent using a VPixx/3D monitor.

EEG data preprocessing was performed using the Matlab-based toolbox Letswave 5 (Mouraux and Iannetti 2008, The MathWorks, Inc., 2022). We first assigned the standard 10-20 system location to the electrodes on the cap (for exact measurements, see www.biosemi.com). A Butterworth bandpass filter (fourth order) was used to perform high- and low-pass filtering of the signal, within the frequency range of 0.1 to 100 Hz. We subsequently downsampled our EEG data to 256 Hz to enhance computer processing speed and segmented them to include 2 seconds before and after each sequence, resulting in epochs with a duration of 66 seconds (ranging from −2 to 64 seconds). Eye movements and blink artifacts were removed using independent component analysis. Electrodes with voltage exceeding 100 μV were interpolated using the three closest neighboring channels. For each participant, no more than 5% of all channels were interpolated. To enhance data quality, all epochs were visually inspected, and the two with the most artifacts were excluded from analysis. Following the cleaning procedure, one participant in Experiment 1 was excluded from further analysis due to insufficient data quality. The final sample comprised 43 participants (*M_age_*= 22.7, *SD_age_* = 3.3, 5 males).

Data were then re-referenced to the common average and segmented to exclude the first two and last two seconds of stimulation—corresponding to fade-in and fade-out—and to include an integer number of oddball cycles. This procedure yielded epochs of approximately 58.3 seconds, comprising 14,933 data points each. Finally, segments were average for each condition (i.e. EA and WE as oddball) and participants separately.

*Frequency domain*. Fast Fourier Transform (FFT) was applied to the averaged segments to extract the amplitude of the frequency spectrum. To assess the statistical significance of the neural response to oddball stimuli, we calculated the Z-scores from the FFT spectra at the fundamental oddball frequency and its harmonics separately for each electrode, participant and race. This was done by subtracting the mean amplitude of the 20 surrounding bins (excluding the two immediately adjacent bins) from the amplitude of the bin of interest and dividing the result by the standard deviation of these bins’ amplitude. These Z-scores were then averaged across participants and race, and used to identify the presence of significant harmonics (i.e. Z-scores larger than 1.64 for two consecutive harmonics, *P* < .05, one-tailed). We examined electrodes at occipitotemporal sites (A9–A16, A21–A29, B6–B11, D31–D32) covering regions most consistently associated with the face-specific N170 ERP component (e.g. Stahl *et al*. 2010, Caharel *et al*. 2011, Senholzi and Ito 2013, Sun *et al*. 2013, Yao and Zhao 2019, de Lissa *et al*. 2024).

*Statistical analysis*. We analyzed behavioral data in R (RStudio 2023.12.1). As residual diagnostics indicated non-normal response times (RT) residuals (Q–Q tail deviations), and non-normality persisted after log transformation, W = 0.63, *P* < .001, RT was analyzed with a Gamma generalized linear mixed-effects models (log link) (lme4; Bates *et al*. 2015). Accuracy was analyzed with binomial generalized linear mixed-effects models (logit link). Fixed within-subject effects included Face Race and scaled/centered Trial Number. Random effects included participant-specific intercepts and participant-specific slopes for Trial Number. Fixed effects in RT models were evaluated with ANOVA using Kenward–Roger degrees of freedom, whereas fixed effects in accuracy models were evaluated using Type-III Wald chi-square tests.

### Results

*Behavioral data.* A significant main effect of race was observed on accuracy, χ^2^(1) = 7.5, *P* = .006. Estimated marginal means indicated that accuracy was higher during sequences with SR (94.7%, 95% CI [92.6%, 96.2%]) than OR oddballs (92.5%, 95% CI [89.9%, 94.5]). Other effects on RT or accuracy were not significant (*Ps* > .05).

**Figure 2:**
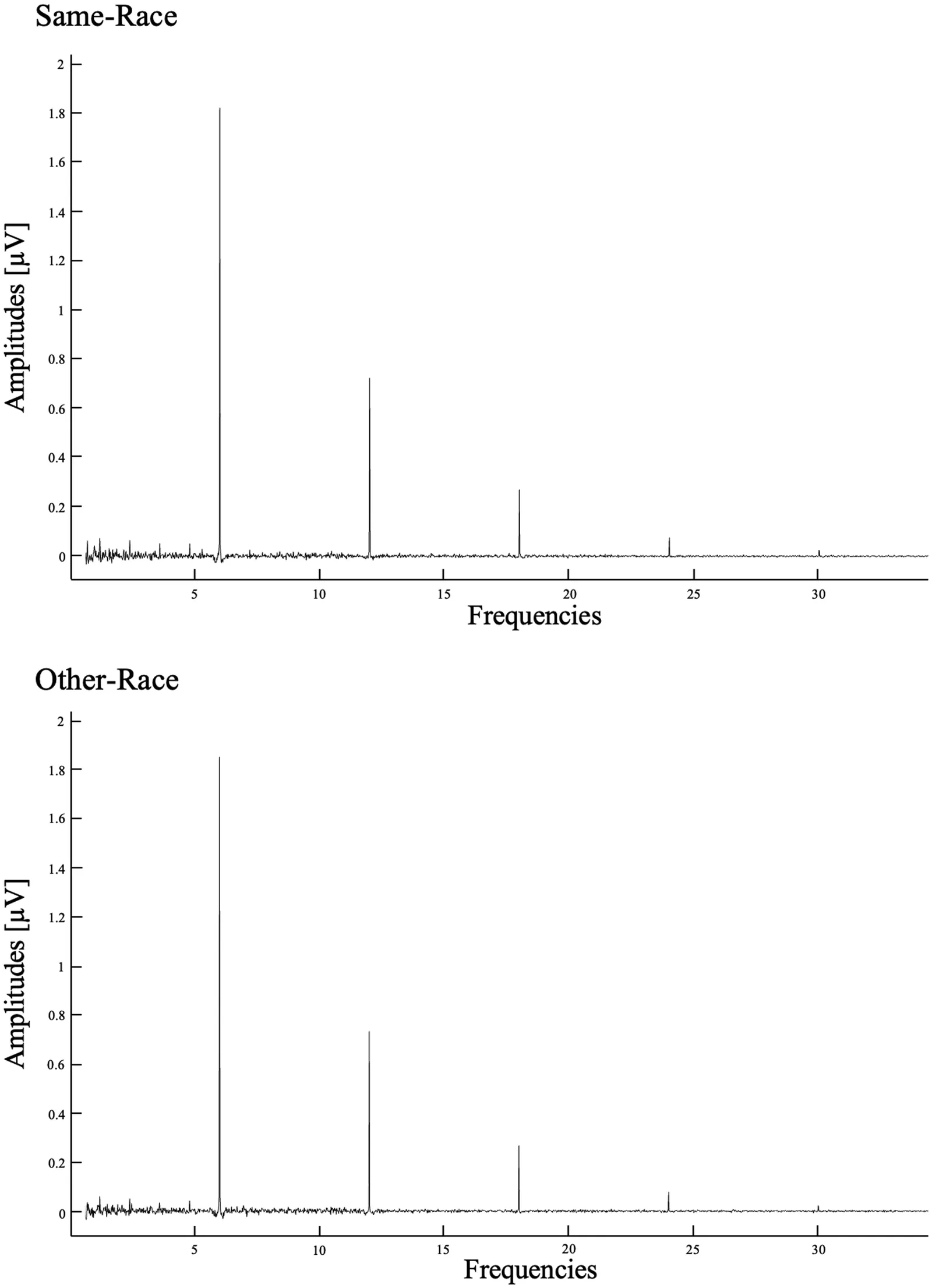
Averaged baseline corrected spectrum for the race discrimination task.

*Neural data*. Z-scores revealed that the neural response at the oddball frequency (fundamental frequency or its harmonics) was not significantly above noise level (Fig. 2, Table 1). Therefore, no further analyses were conducted on these data.

**Table 1:** Mean (*M*) and standard deviation (*SD*) of Z-scores and baseline-corrected amplitudes at the oddball frequency (1.2 Hz) and its non-base-rate harmonics (2.4, 3.6, and 4.8 Hz) for East Asian (EA) and Western European (WE) oddball conditions (*N *= 43 per condition).

Z scores				Baseline corrected spectrum	
Race	Frequency	*M*	*SD*	*M*	*SD*
**EA**	1.2	0.53	1.1	0.06	0.12
	2.4	0.66	1.07	0.05	0.08
	3.6	0.59	0.88	0.04	0.05
	4.8	0.76	1.08	0.04	0.06
**WE**	1.2	0.69	0.98	0.07	0.12
	2.4	0.87	1	0.07	0.07
	3.6	0.83	0.87	0.05	0.05
	4.8	0.9	1.05	0.05	0.07

## Experiment 2—face categorization and impact of inversion

Experiment 2 explored the neural categorization responses of SR and OR faces from objects. To assess the impact of stimulus inversion on race-related neural response, Experiment 2 was conducted once with all stimuli presented upright and once with all stimuli inverted.

### Methods

#### Participants

The same participants as in Experiment 1 took part in Experiment 2.

#### Materials

A total of 211 non-face objects previously used in other oddball-FPVS paradigms (de Heering and Rossion 2015, Rossion *et al*. 2015, Yildirim-Keles *et al*. 2025) were added to the face sample. They matched the dimensions of the face pictures (320 × 320 pixels), were also equalized for luminance and contrast, and varied in terms of position and background. The pictures could represent living entities or parts of living entities (e.g. plants, fruits, *N *= 82), or inanimate items (e.g. houses, built objects, *N *= 129). The computer and software were the same as in Experiment 1.

#### Procedure

**Figure 3:**
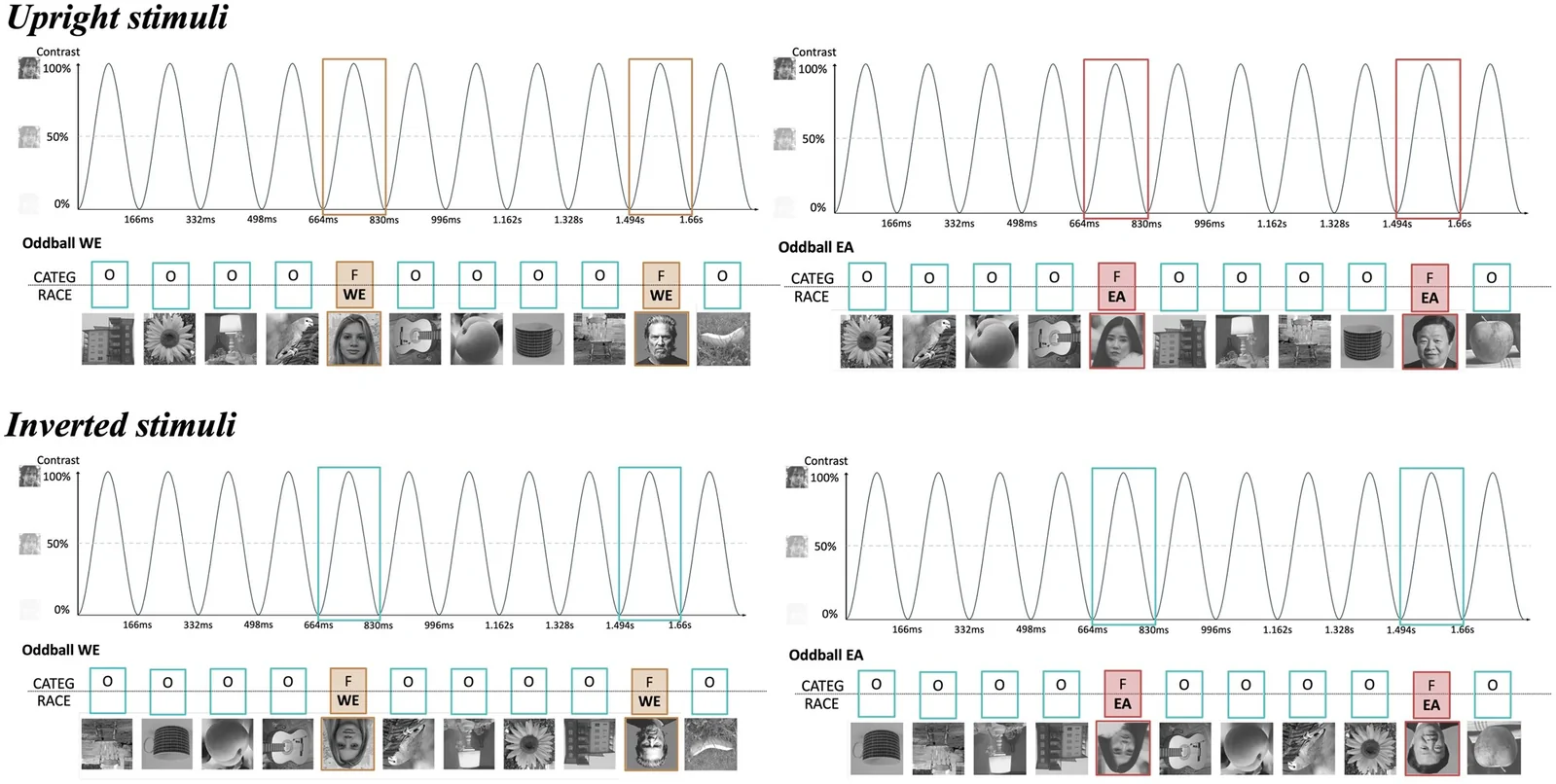
The paradigm of the oddball-FPVS face categorization task with upright and inverted stimuli.

The frequency of visual stimulation and number of trials in Experiment 2 were the same as those reported for Experiment 1. However, while as in Experiment 1, faces from either race were presented as oddballs, here base stimuli consisted of images of non-face objects (Fig. 3). Eight trials with upright stimuli (four trials per race) were followed by eight trials with inverted stimuli (lasting approximately 25–30 minutes in total). The stimuli in each trial were presented following the same semi-randomized procedure as in Experiment 1.

#### EEG acquisition and preprocessing

The EEG system, recording settings and pre-processing steps were the same as those reported for Experiment 1. Due to excessive artifacts in the signal, two additional participants (three in total from the original sample) were excluded from formal analysis, resulting in a final sample size of 41 participants (*M_age_* = 22.8, *SD_age_* = 3.3, 5 males).

**Figure 4:**
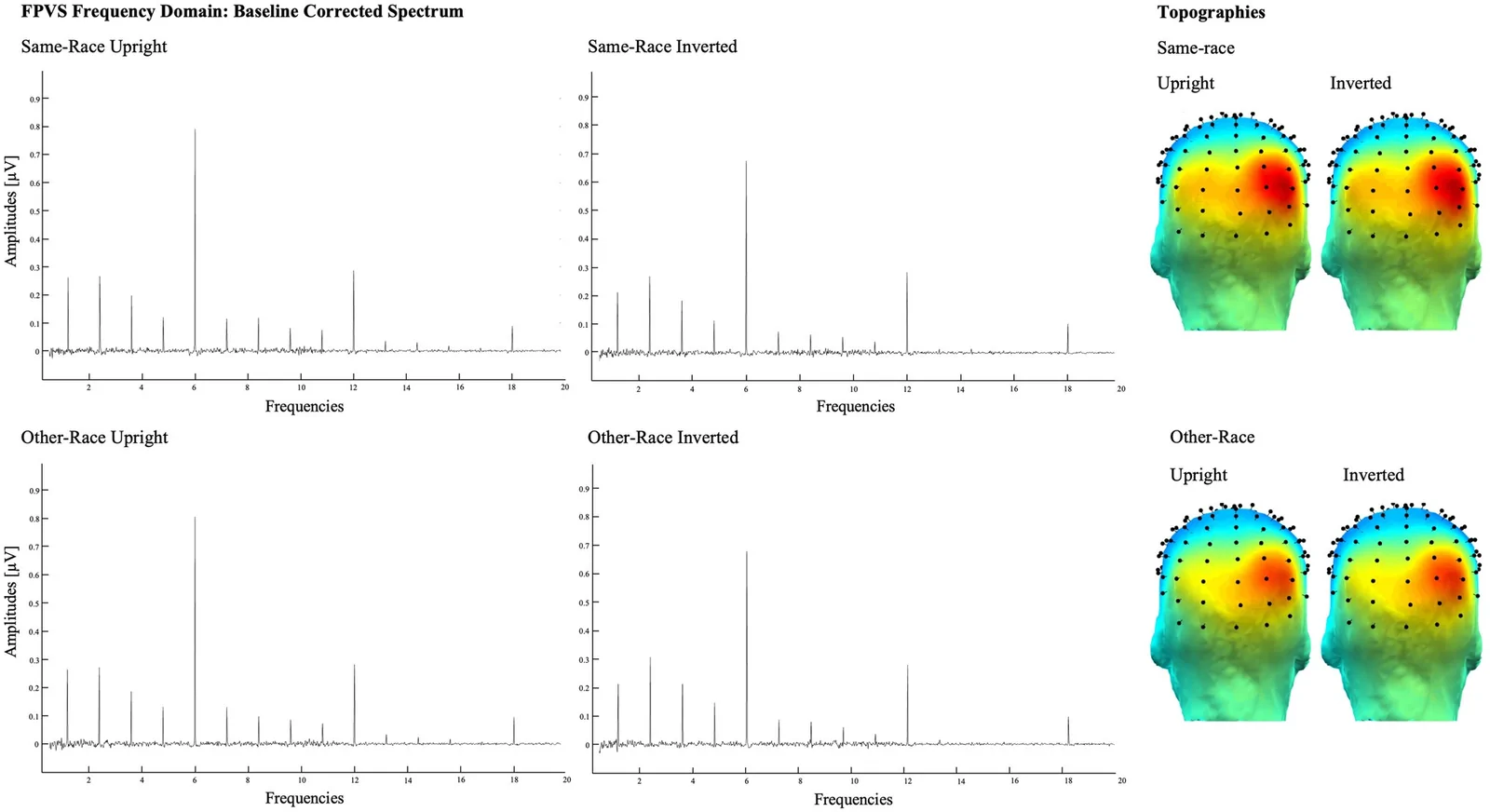
Averaged baseline corrected spectrum for the face categorization tasks and topographies of raw voltage.

*Frequency domain.* As for Experiment 1, FFT was applied and Z-scores were computed on the FFT spectra of each electrode and subsequently averaged across participants, race and orientation. Significant responses (Z-scores larger than 1.64 in occipitotemporal channels, *P* < .05) were observed at the oddball frequency (1.2 Hz) and 9 of its harmonics (i.e. 2.4 Hz, 3.6 Hz, 4.8 Hz, 7.2 Hz, 8.4 Hz, 9.6 Hz, 10.8 Hz, 13.2 Hz, and 14.4 Hz). Amplitudes were baseline corrected by subtracting, from each frequency bin, the mean amplitude of the 20 surrounding bins (excluding the two adjacent bins) (Fig. 4). Baseline-corrected amplitudes were summed across significant oddball frequencies for each face race (SR or OR) × orientation (upright or inverted) combination.

*Statistical analysis.* Behavioral data were analyzed with the same software (RStudio 2023.12.1) and tests (Gamma generalized linear mixed-effects models with ANOVA and binomial generalized linear mixed-effects models with chi-square tests) as for the race discrimination task. However, orientation was included as an additional within-subject fixed effect.

**Figure 5:**
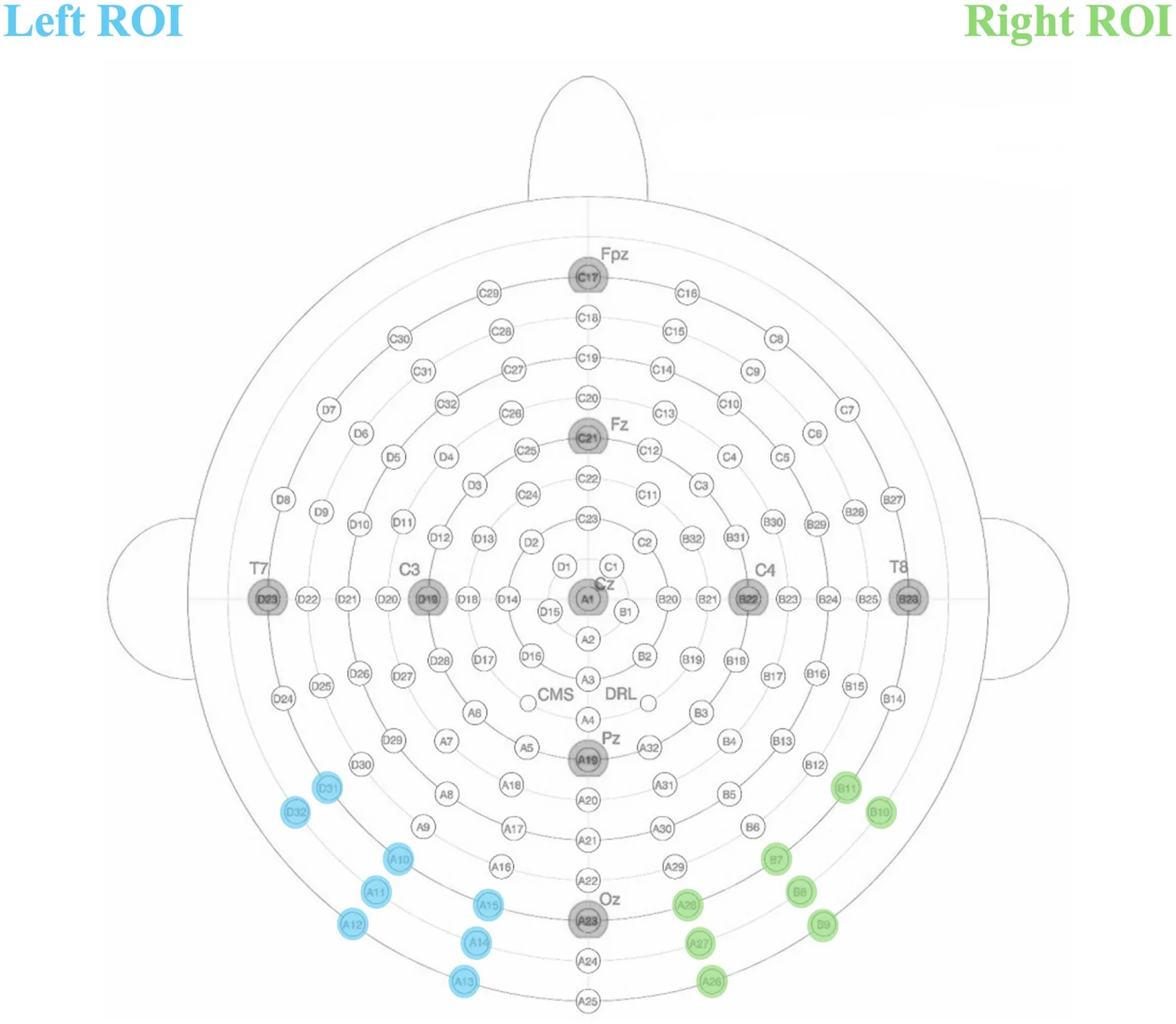
Representation of the ROIs on the EEG 128-electrode system (Biosemi). The green and blue markers indicate the electrodes selected for the left and right ROIs, respectively.

Neural data analyses were performed using RStudio 2023.12.1. We fitted a linear mixed-effects model using the lme4 package (Bates *et al*. 2015). The model included face race, orientation, and ROI as fixed within-subject factors, with participants as random factors for race, orientation, and ROI. The significance of the fixed effects was assessed using an ANOVA, with degrees of freedom estimated with the Kenward–Roger method. Post hoc contrasts were corrected for multiple comparisons using the Tukey method. The analyses were performed within two occipitotemporal regions of interest (ROI): a left ROI (encompassing electrodes A10–A15 and D31–D32) and a right ROI (A26–28 and B7–11), encompassing the electrodes P7, P9, PO7, PO9, PO11, O1, POI1, I1 and O2, POI2, I2, PO8, PO10, PO12, P8, P10 in the 10-20 system (see Rossion *et al*. 2015), respectively (Fig. 5).

### Results

*Behavioral data*. Accuracy showed a significant Race x Orientation interaction, χ^2^(1) = 4.38, *P* = .04. Holm-corrected simple-effects comparisons at the centered trial value (TrialNumber_z = 0) showed that accuracy in fixation color’s detection was higher for SR oddballs (95.1%, 95% CI [93.6%, 96.3%]) than OR oddballs (93.6%, 95% CI [91.8%, 95.1%]) in the upright condition, *P* = .048. In contrast, no Race difference was observed in the inverted condition (SR = 93.5%, 95% CI [91.6%, 95.0%]; OR = 94.2%, 95% CI [92.5%, 95.6%]), *P* = .33. The effect of Orientation was significant during sequences with SR oddballs, *P* = .03, but not with OR ones, *P* = .43. No other effects reached significance for accuracy or response times in either the race discrimination or face categorization tasks (all ps > .05).

**Figure 6:**
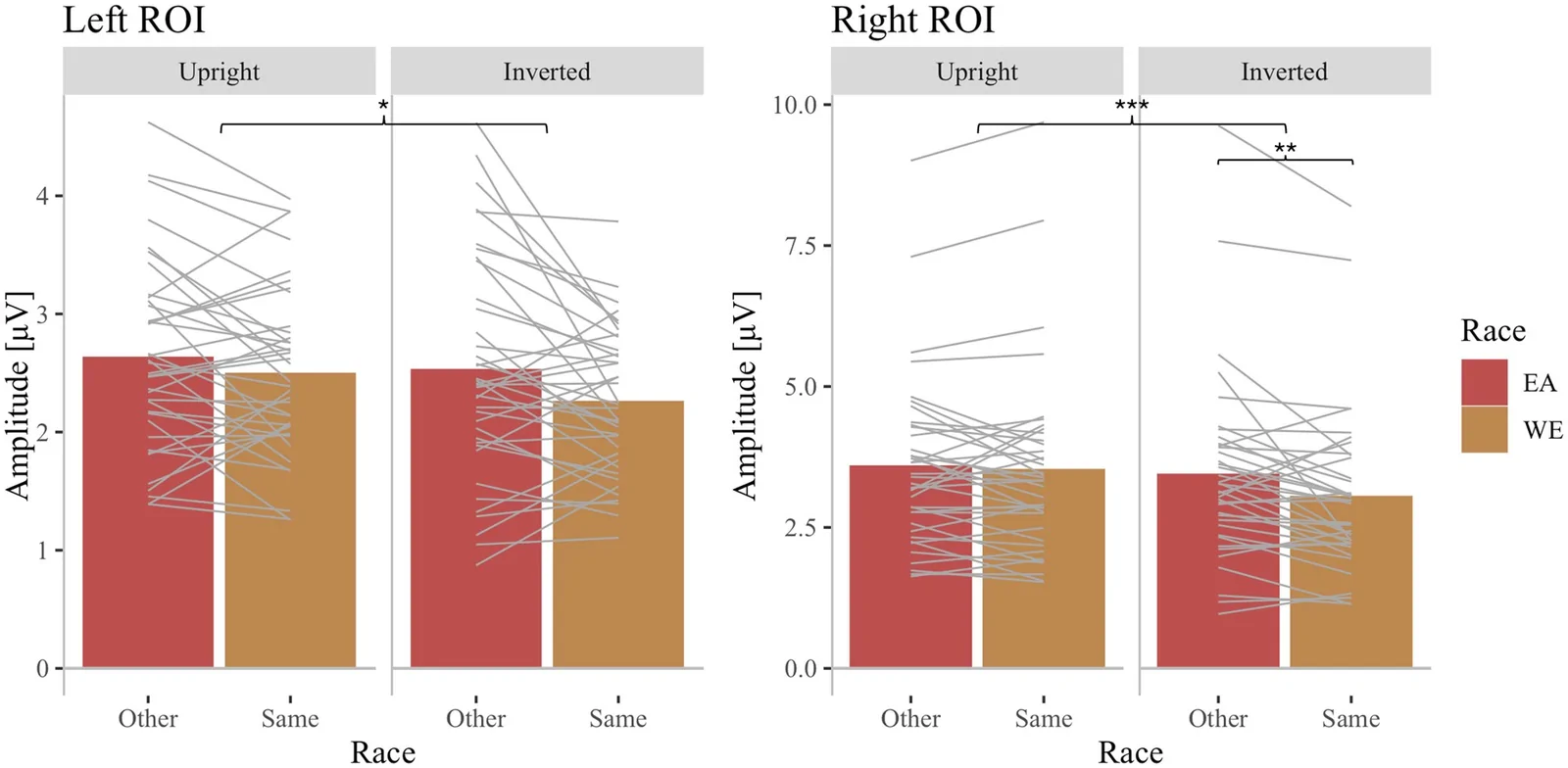
FPVS response amplitude in two regions of interest for upright and inverted face categorization tasks. *P < .05, **P < .001.

*Neural data*. The ANOVA revealed a main effect of Race, *F*(1, 40) = 10.33, *P* = .003, η^2^ = .21, with greater response amplitudes for OR (*M *= 3.2, *SD *= 1.6) than for SR oddballs (*M *= 3, *SD *= 1.5) (Fig. 6). There was also a main effect of Orientation, *F*(1, 40) = 4.63, *P* = .04, η^2^ = .1, with greater response amplitudes for Upright (*M *= 3.2, *SD *= 1.6) than Inverted stimuli (*M *= 3, *SD *= 1.4). Additionally, a significant main effect of Hemisphere was observed, *F*(1, 40) = 17.84, *P* < .001, η^2^ = .31. Response amplitudes were larger in the right (*M *= 3.6, *SD *= 1.9) than in the left ROI (*M *= 2.5, *SD *= 0.8). The interaction of Race × Orientation also reached significance, *F*(1, 160) = 5.43, *P* = .02, η^2^ = .03. Post hoc contrasts revealed that, in the upright condition, oddball response amplitudes were similar for OR (*M *= 3.2, *SD *= 1.7) and SR oddballs (*M *= 3.1, *SD *= 1.6), *P* = .21, *d* = .23. In contrast, when stimuli were inverted, SR oddballs (*M *= 2.8, *SD *= 1.3) led to a weaker neural response than OR ones (*M *= 3.1, *SD *= 1.5), *P* < .001, *d* = .75. This pattern of response was driven by the inversion, which significantly reduced the response to SR oddballs (*P* = .006, *d* = .83), but not to OR ones (*P* = .28, *d* = .32). None of the other interaction effects reached significance (all *P* > .08).

## Discussion

This study aimed to investigate rapid neural face categorization responses to same-race (SR; Western European) and other-race (OR; East Asian) faces using an oddball fast periodic visual stimulation (FPVS) paradigm and examined how these responses are modulated by stimulus inversion. In Experiment 1, we assessed neural race discrimination by presenting faces of a specific race as base stimuli, while faces of the alternative race served as oddballs. Experiment 2 explored neural face categorization responses to SR and OR faces using non-face objects as base stimuli and faces of either race as oddballs. To investigate the impact of stimulus orientation on rapid categorization, the sequences of stimuli were presented either upright or inverted.

Results from Experiment 1 showed that oddball response during neural face race discrimination was not significantly above noise level for either face race. In contrast, in Experiment 2, we found significant oddball responses to both face races during rapid neural face categorization. Accordingly, subsequent analysis exclusively focused on data collected during the latter experiment. Results revealed that rapid neural face categorization was stronger for OR compared to SR faces, for Upright than Inverted stimuli, as well as in the right relative to the left Hemisphere. Additionally, we found a significant interaction between face race and orientation. Specifically, while neural responses to SR and OR faces were comparable in the upright viewing condition, stimulus inversion selectively reduced responses to SR faces, with no such reduction observed for OR faces. Finally, the behavioral results revealed a higher detection rate of fixation color changes during sequences containing SR oddballs compared to those with OR oddballs. This effect was observed in both the race discrimination task and the face categorization task with upright stimuli, but it disappeared when the stimuli were inverted. This pattern was driven by a significant orientation effect that reduced performance during SR oddball sequences, whereas performance during OR oddball sequences was not affected by inversion.

### Behavioral results

The superior performance observed during upright SR oddball sequences in the orthogonal task may reflect differential attentional capture by different face races (Schaller and Caldara, in press). This aligns with previous research suggesting that categorization by race occurs more automatically for OR than SR faces (Duncan *et al*. 2022), and that OR faces can unconsciously guide visual attention (Hu *et al*. submitted) and capture attention more strongly than SR faces (Al-Janabi *et al*. 2012). Specifically, during upright face categorization, greater attentional resources to OR oddball faces might have left comparatively fewer resources available for the color detection task. Familiarity with a stimulus category may also explain why performance on the orthogonal task decreased with inversion but no longer differed across face races. It is possible that inversion causes both face races to become equally unfamiliar. Consequently, inverted SR oddball faces may capture more attention than when they are presented upright, matching the unfamiliarity level of OR oddball faces.

According to this explanation, for the face race discrimination task, one might initially expect, contrary to our findings, that sequences with a greater proportion of OR faces would disrupt performance at the orthogonal task to a greater extent than sequences with more SR faces. However, we suggest that familiarity remains the most plausible explanation in this scenario as well. Because OR faces belong to an unfamiliar face category, individual identities within OR faces are less distinctive, a hallmark of the Same-Race Recognition Advantage (Valentine 1991, Singh *et al*. 2022). By presenting multiple consecutive OR faces during SR oddball sequences, it is possible that their higher perceptual homogeneity reduced their overall visual saliency and therefore their impact on attentional distribution. On the other hand, when OR faces are presented as oddball among SR faces, their infrequent appearance coupled with their unfamiliarity makes them highly distinctive. This is consistent with results from face detection paradigms where out-group faces act as highly salient visual features that more readily capture attention than SR faces (e.g. Levin 1996, 2000).

It is crucial to note that these explanations require further research. This is especially important because, although the reported effect is significant, the effect size is small, and participants reach ceiling performance across all conditions. Nevertheless, these results suggest that differences in neural responses cannot be accounted for by performance on the orthogonal task, as greater color detection performance is not reflected in stronger oddball responses.

### Face race discrimination

The lack of a neural race discrimination response in Experiment 1 requires explanation. At first sight, one might hypothesize that rapid stimulus presentation combined with high visual variability interfered with race processing. However, prior evidence argues against this interpretation. Race-related information is processed rapidly and efficiently under challenging conditions, including rapid presentations (100 ms, Caldara *et al*. 2004), peripheral vision (de Lissa *et al*. 2021), and visual noise (de Lissa *et al*. 2022, 2024), suggesting that our 166 ms stimulus duration was sufficient for neural race discrimination.

We instead propose that the null result reflects inherent FPVS properties. Oddball-FPVS requires reliable differential neural responses between base and oddball stimuli to generate a detectable frequency-domain peak. Because all stimuli in Experiment 1 were faces, which evoke strong neural responses (Bentin *et al*. 1996), the periodic oddball response likely overlapped in amplitude and topography with the base face response. This overlap prevented emergence of a distinct oddball-specific peak, suggesting that neural processing differences based on race were too subtle to produce a separable response, particularly given our use of complex natural images rather than simplified stimuli. However, further studies are necessary to disentangle whether the results we observed here are due to an insufficient signal to noise ratio or a genuine lack of sensitivity to race with this paradigm.

### Face categorization

In Experiment 2, the stronger contrast between the neural response evoked by objects and faces elicited a clear oddball-specific response, with significant peaks above noise level in the frequency spectrum. This finding is in line with prior work using FPVS, which found significant neural categorization of SR faces from non-face objects in both infants (de Heering and Rossion 2015) and adults (Rossion *et al*. 2015) and extends them by showing that OR faces are also efficiently discriminated from non-face objects. Based on these observations, we compared the strength of the oddball response across face races, orientations and hemispheres.

Larger oddball responses were observed in the right relative to the left hemisphere. This hemispheric asymmetry may be related to differences in face-processing specialization. A substantial body of literature recognizes the right hemisphere as the dominant locus for face processing, consistently exhibiting stronger face-selective responses relative to the left hemisphere (Behrmann and Plaut 2020, Lesinger *et al*. 2023). Importantly, this main effect of ROI was not characterized by an interaction with Race, suggesting that hemispheric lateralization was comparable across face categories. This interpretation is consistent with studies reporting that race processing relies on a broad and distributed neural network (Schaller *et al*. 2023, 2025). Additionally, using an FPVS paradigm, Wallsinger *et al*. (2025) recently demonstrated that neural face identity discrimination becomes increasingly right lateralized throughout infancy and is not impacted by race in infants. These findings suggest that right-hemisphere dominance for face individuation can be observed in the absence of race-related modulation. Our results show that this pattern may also emerge during face categorization in adults, where we observed a larger oddball response in the right hemisphere that was not modulated by race.

The results also showed a stronger response amplitude to OR than SR oddballs. Importantly, this main effect of Race was qualified by a significant Race × Orientation interaction. Specifically, response amplitudes did not significantly differ between SR and OR oddballs in the upright condition, whereas a significant difference emerged in the inverted condition. This effect was driven by a selective decrease in response amplitude to SR faces, while responses to OR faces remained stable. Because FPVS and ERP index different responses (Yildirim-Keles *et al*. 2025) and the paradigms differed with previous electrophysiological studies (i.e. faces shown in isolation vs. embedded in an object stream), a direct comparison is precluded. Nevertheless, these results align with ERPs findings showing stronger inversion effect on the P100 (Colombatto and McCarthy 2017) or N170 (Vizioli *et al*. 2010, Wiese 2013) for same- than other-race faces. Additionally, at the behavioral level, inversion disproportionately disrupts recognition of objects categories with which individuals developed expertise as compared to less familiar categories, suggesting that the FIE may serve as a general marker of expertise-related processing (Diamond and Carey 1986, Campbell and Tanaka 2018, Gahl *et al*. 2024). As such, the neural FPVS responses observed for inverted stimuli might relate to a marked visual expertise for same- compared to other-race faces.

Regarding more specifically the race-related difference in response amplitudes in the inverted condition, although our findings are limited to a single cohort of Western European participants, which restricts immediate generalizability across cultures, they show that FPVS can successfully capture race-related neural differences. Importantly, differences in physical stimulus properties are unlikely to fully account for the observed effect. First, luminance and contrast were matched between set. Second, the difference in physical homogeneity between the SR and the OR stimuli sets was negligible (Cohen’s *d *= 0.11). And third, the same face set did not elicit a significant oddball response in medial electrodes in Experiment 1, likely because the low-level visual differences between face races were too subtle to elicit a measurable distinct neural response. Additionally, these findings are partially in line with Vizioli *et al*. (2010) who found race-related modulation of the N170 for inverted but not upright faces. However, this study measured transient ERPs to spatially isolated face stimuli, whereas the present study specifically examined the neural categorization of faces from non-face objects. Our results are also consistent with fMRI studies showing race-related activations of the FFA and/or OFA (Golby *et al*. 2001, Feng *et al.* 2011, Natu and O’Toole 2011, Golarai *et al*. 2021). A possible interpretation for our findings is that the OR advantage driven by the greater saliency of OR faces (Levin 1996, 2000, Thornton *et al*. 2019, de Lissa *et al*. 2021) might be masked in the upright condition by human’s proficiency in face processing (Behrmann and Plaut 2020, Lesinger *et al*. 2023), and only become apparent under more challenging viewing condition, such as when stimuli are presented inverted. Altogether, our data show that, while visually demanding, FPVS can capture race-related neural differences. The detection of race in the context of a rapid visual stream of objects and faces highlights the sensitivity of this electrophysiological technique and paradigm to socially relevant visual cues, even under ecologically valid conditions reflecting real-life diversity. Stronger neural responses to other-race faces may reflect their heightened perceptual salience and the greater automaticity of categorization processes, regardless of stimulus orientation.

## Conclusions

This study is the first to use FPVS to investigate race effects in face categorization rather than transient ERPs to isolated faces. Our findings suggest that, in Western European participants, while race-related cues may not always elicit robust differential neural responses to SR vs. OR faces during rapid face-only streams under naturalistic conditions, such differences may emerge when faces are contrasted with object bases. Importantly, race did not modulate FPVS response amplitude in the upright condition, yet inversion uncovered differential SR and OR neural responses, highlighting the role of expertise in shaping perceptual mechanisms. As inversion uncovered race-related modulations that were absent in the upright condition, it would be of interest for future FPVS studies to investigate whether inverting faces would elicit significant race discrimination. Overall, the data support frequency-tagged EEG as a sensitive approach to studying race-selective and broader social categorization mechanisms, complementing insights from functional neuroimaging and opening new research avenues.

## Data Availability

Data used in this study are available on the Open Science Foundation (OSF) repository at https://osf.io/wbrny/overview? view_only=238eeb1d3f614de38dc5afd5ad1c1b4a
